# A Serendipitous Outcome of COVID-19: Modifications to ICU Management

**DOI:** 10.21315/mjms2023.30.6.3

**Published:** 2023-12-19

**Authors:** Syeda Warda Murshad, Arsalan Aamir Khan, Suraksha Rani, Muhammad Kamran

**Affiliations:** 1 Dow University of Health Sciences, Karachi, Pakistan; 2 Jinnah Medical and Dental College, Karachi, Pakistan

**Keywords:** intensive care units, healthcare-associated infections, COVID-19, critical care

## Abstract

Nosocomial infections are common in intensive care units (ICUs) and often cause increased morbidity and mortality rates in ICU patients. With the emergence of the highly infectious COVID-19, the high prevalence of hospital-acquired infections (HAIs) in ICU has caused much more concern because patients admitted to the ICU have a more severe and prolonged form of the disease. These patients are more likely to develop HAIs than non-ICU patients. Medical communities adopted several measures to make ICU management safer during the pandemic all over the world. In this study, we re-examined the challenges faced and the changes made in ICU management during the pandemic to speculate how these changes will be relevant post-pandemic and can be permanently incorporated into the ICU to improve safety, management, and critical care and make critical care better equipped for future disease breakouts.

## Introduction

Hospital-acquired infections (HAIs) are common in intensive care units (ICUs) and often cause increased morbidity and mortality rates in ICU patients (1). A prevalence survey before the pandemic reported a high incidence of HAIs in ICUs, ranging from 23% to 9.1% in first-world countries and even more (35.2%) in lower-middle-income countries (LMICs) (2). With the emergence of the highly infectious COVID-19, the high prevalence of HAIs in the ICU has caused much more concern because COVID-19 patients admitted to the ICU have a more severe and prolonged form of the disease. These patients are more likely to develop HAIs than non-ICU patients (1). Several factors play a role in the development of ICU infections, including immune-suppressing steroids, chemotherapy, indwelling catheters, aerosol-generating procedures (AGPs) and prolonged ICU stays (1). ICU staff and physicians can also act as carriers of infectious agents from other wards to the ICU or vice versa (1). Variations in standard infection control practices stem from a lack of evidence-based infection control recommendations for ICU-infected COVID-19 patients (3). In this study, we examined ICU management problems that emerged during the COVID-19 pandemic as well as changes made in ICU management during the pandemic to optimise infection control to speculate how these changes will be relevant post-pandemic and can be permanently incorporated into the ICU and limit the spread of HAIs.

### COVID-19: ICU Management Challenges

During the ongoing COVID-19 pandemic, it became clear that the ICUs were inadequately designed and equipped to treat patients infected with highly contagious viruses, such as severe acute respiratory syndrome coronavirus 2 (SARS-CoV-2) (4).

AGPs were quickly associated with an increased risk of HAIs (5). According to the WHO, bronchoscopy, tracheotomy, non-invasive positive pressure ventilation, cardiopulmonary resuscitation, intubation and sputum induction are some examples of AGPs (6). According to other professional societies, nasogastric tube insertion, thoracentesis, colonoscopy, esophagogastroduodenoscopy, cardiac catheterisation, exercise tolerance tests, pulmonary function tests, percutaneous gastric tube insertion, facial surgery and the second stage of labour are considered AGPs (6). However, it is unclear which procedures produce aerosols in practice (6). Currently, high transmission risk is attributed to prolonged and close contact with patients with a high viral load during these procedures rather than to a specific procedure itself. A patient experiencing symptoms is more likely to have an active infection. Because the concentration of respiratory emissions is highest near the source of infection, such patients, in case of close contact with them, increase the risk of infection transmission through coughing or sneezing (6).

Invasive procedures performed during hospital stays put ICU patients at the highest risk for HAIs (1). AGPs, chemotherapy, indwelling catheters and other procedures are some examples of invasive procedures (1). Critically ill COVID-19 patients were mostly older men with multiple comorbidities, such as hypertension, diabetes and chronic obstructive pulmonary disease (COPD), who had severe respiratory failure and prolonged duration of disease. Most of these patients were treated in ICUs and received invasive mechanical ventilation (90%) as an AGP (3) and immunosuppressive agents; therefore, they were at higher risk of developing HAIs (3).

In addition, contact of the patients with healthcare workers (HCWs) strongly contributes to the spread of nosocomial pathogens. Nurses and hospital housekeeping staff are considered the most contaminated hospital staff because they often contaminate their hands during direct patient handling or due to contact with body secretions and contaminated surfaces in the hospital, especially the ICU (1). During the COVID-19 pandemic, this issue was a major deterrent to infection control as the hospitals were understaffed and ICUs were overwhelmed with an unprecedented increase of critically ill patients making HCWs attending to more than one ward or ICU, a route of transmission of COVID-19 and HAIs (1).

Moreover, central air conditioning increases the incidence of HAIs in both infected and non-infected patients, as the air exhaled by patients in ICUs and COVID-19 wards is recirculated into the room, which increases the chance of transmission and the severity of infection in those who are already infected (4). More than 500 doctors, nurses and other HCWs have died due to COVID-19 worldwide while serving patients in ICUs around the world (4), implying that ICUs should be modified to reduce the risk of exposure.

## Methods

A narrative review was conducted by an extensive literature search in PubMed and Google Scholar using keywords, including ‘Intensive Care Unit,’ ‘Healthcare-Associated Infections,’ ‘COVID-19’ and ‘Critical Care.’ We searched for studies published from December 2020 to January 2022 on healthcare-associated infections in COVID-19 patients receiving critical care in ICUs.

## Discussion

Medical communities adopted several measures to make ICU management safer during the pandemic worldwide. Antibiotics, particularly azithromycin, which belongs to the Macrolide class, were given to patients who had a severe COVID-19 infection. This medication has an anti-inflammatory effect on the airways, in addition to helping in controlling and preventing secondary bacterial infections (7). In addition, high-flow nasal cannulas (HFNC) were used for mild to moderate COVID-19 infections. These were found to be more beneficial than regular oxygen because they reduced the likelihood of intubation and treatment escalation. This beneficial effect is most likely due to its ability to meet patients’ needs for respiratory airflow, reduce the effort required to inhale and reduce the risk of self-inflicted lung injury. The heat and humidity of the HFNC also aid in secretion mobilisation, benefiting COVID-19 patients through mucus hypersecretion (8).

According to the Indian Society of Critical Care Medicine (ISCCM), all ICUs serving critically ill patients have a filtration efficiency of 99% for particles larger than 5 mm (4). For smaller particles, ICUs have negative pressure (4). Furthermore, each critically ill COVID-19 patient should be placed in a separate room in the ICU (4). Due to the devastating impact of the COVID-19 quarantine on the global economy, this action was expensive and impossible (4). Designating a well-ventilated structure or a non-air-conditioned hall during the construction of ICUs and makeshift hospitals became a cost-effective and practical alternative (4). In pre-existing ICUs, it was considered appropriate to turn off the air conditioners with all windows open to prevent the recirculation of air exhaled by infected patients (4). Exhaust and air blow fans were also recommended to allow to force the airflow in one direction (4). Soap and sodium hypochlorite could be used to remove air bubbles because they destroy the structural integrity of the virus (4).

Several public health organisations also suggested a higher level of protection during an AGP (5). According to experts, for aerosol-generating treatments, specially constructed or designated rooms, such as airborne infectious isolation rooms (AIIRs), should be considered in the ICU (9, 10). These AIIRs are maintained at a negative pressure relative to the outside environment and allow air to flow into the isolation room but not out. A high-efficiency particulate air (HEPA) filter is also attached to the gas outlet connected to the atmosphere, allowing air to be filtered and exchanged at least 6–12 times per hour (10–12). In addition, the COVID-19 ICUs and their air conditioning vents should be separated from other ICUs and far away from high-risk areas, such as the neonatal intensive care unit (NICUs), delivery rooms, dialysis units and post-operative surgical units (4). Therefore, the equipment and the personnel of COVID-19 ICUs should be separated from other parts of the hospital, allowing for better infection control (12).

Moreover, surgical masks alone were considered sufficient for controlled procedures on asymptomatic patients in areas with a low prevalence of SARS-CoV-2 infections. However, a higher level of respiratory protection, such as face shields, was required for HCWs in high-incidence areas where they should be close to patients’ respiratory tracts (6). Using disposable equipment was encouraged wherever possible. Regarding non-disposable equipment, such as stethoscopes, blood pressure cuffs, thermometers and oximeters, cleansing with 70% ethyl alcohol or quaternary ammonium compounds was recommended (13). Surface disinfection is also an integral part of infection control (13). Thus, surfaces are categorised into two types: i) high-touch and ii) low touch surfaces (13). Doorknobs, bed rails, light switches, toilet walls and privacy curtain edges are all high-touch surfaces that should be frequently disinfected (13). Floors, ceilings, walls, curtains and blinds are low-touch surfaces that require less frequent cleaning; usually twice a day and a damp mop is preferred over dry cleaning (13). Besides disinfecting equipment and surfaces, corridors used for patient transport should also be cleaned regularly (8). Sodium hypochlorite is recommended as the ideal agent for cleaning surfaces during the patient stay and after discharge due to its broad-spectrum antibacterial effect (14).

Personal protective equipment (PPE) in hospitals was undoubtedly of utmost importance in the midst of the pandemic. However, in addition to appropriate PPE, adequate space for donning and doffing PPE was critical for each ICU to reduce the risk of cross-contamination (4). A donning area is a ‘clean filter’, whereas a doffing area is a ‘contaminated filter’ (13). Both spaces should have waste management containers and an appropriate area with sanitiser for hand washing; however, the donning area should also have disposable PPE and surgical scrubs (13). To further limit the risk of disease transmission, all hospital staff practiced hand hygiene and showered before leaving the premises after removing their scrubs and PPE (13). A mechanism called the ‘buddy system’ was implemented in many places to ensure that all HCWs followed these guidelines (13). The buddy system forms groups of two or more HCWs, in which the group members share responsibility for each other’s safety by ensuring these precautions are followed (13).

Furthermore, it was necessary to check the temperature of hospital staff regularly and screen for COVID-19 from time to time (15). The HCWs were required to measure and report their body temperature twice a day. This information was collected and analysed to aid in the early detection of infections and contact tracing (15). In addition, the pandemic also required that the potential of telemedicine be fully exploited. Telemedicine avoids unnecessary contact with COVID-19 patients and overcrowding hospitals by remotely monitoring them. Employing elderly physicians in telemedicine relieves the burden on field physicians while utilising the extensive experience and expertise of senior physicians without putting them at risk of exposure (16, 17). Table 1 summarises the measures taking during COVID-19 pandemic in handling patients with COVID-19 infection.

Finally, to limit the transmission of the SARS COV-2, hospitals should ensure that they have the facilities and infrastructures for rapid SARS-COV-2 testing (18). If an individual tests positive, he/she should not be allowed to meet non-infected or suspected COVID-19 patients (4). Ideally, hospitals should have separate entrances, exits, elevators and stairs for the isolation wards and COVID-19 ICUs, with a proper and fully functional security system to prevent unwanted visitors from entering (4). For ICUs, visitor policies were also revised during the pandemic. People with weakened immune systems, such as the elderly or people with multiple comorbidities and those showing symptoms of acute respiratory illness, were not allowed to enter (19), and only those essential to providing patient care were allowed to visit. After entering the ICU, these visitors had to wear a face mask and observe proper hand hygiene (19).

## Conclusion

### Lessons Learned from the Pandemic

The world is now evolving with COVID-19 (20). The pandemic put a significant strain on the intensive care system of hospitals. The COVID-19 pandemic showed that most ICUs were neither designed nor equipped to deal with outbreaks of airborne infectious diseases (4). Several deficiencies were identified in these systems, and numerous changes were made and implemented.

Due to the high prevalence of severe hospital-acquired respiratory illnesses along with the ongoing risk of nosocomial transmission of COVID-19 to critical and often immunocompromised ICU patients (1) (2), it is necessary to make ICU management safer in the future by permanently implementing some mitigation strategies introduced during the pandemic, especially in LMICs with the highest prevalence of HAIs (2) and slow rate of vaccination (20).

Moreover, according to the detailed analysis by Ben Oppenheim and Nicole Stephenson on the epidemic frequency and its global distribution, the spread of infectious diseases from wild animals to humans has steadily increased in frequency and severity. Their results showed that the world might face another pandemic much sooner than expected and estimated a 47%–57% chance of another pandemic as deadly as COVID-19 in the next 25 years. These distressing findings mean that the world and its healthcare systems must be prepared and better equipped for future outbreaks (21). Therefore, learning from the lessons of the COVID-19 pandemic, the authors here suggest strategies for permanent incorporation into ICU management protocols.

After the pandemic, all ICUs should be equipped with a filtration efficiency of 99% for particles larger than 5 mm (about 0.2 in.) and maintain negative pressure. Temporary ICUs with open ventilation and turning off the air conditioners are generally not possible. Therefore, to avoid concentrating pathogenic particles in an air-conditioned ICU, a cheap and feasible alternative is the permanent installation of exhausts and blowers for unidirectional airflow with soap and sodium hypochlorite to clear pathogens from exhaust air bubbles.

Ideally, converting the ICU design from a ward type to a single room type would improve infection control. These rooms can be separated from the nursing station by glass doors to ensure undeterred monitoring (22). However, if this is too expensive, at least AIIRs should be built for AGPs. Such rooms are maintained at a negative pressure and connected to the outside atmosphere with HEPA filters that filter and exchange air 6–12 times per hour. The ICUs designed like a ward should try and perform most AGPs in these AIIRs to limit the contamination of ICUs with pathogen-laden aerosols.

Moreover, during the pandemic, COVID-19 ICUs were separated from non-COVID-19 ICUs. After the pandemic, COVID-19 ICUs can be converted into infectious disease ICUs, which can effectively protect critical and often immunocompromised ICU patients from patients with transmissible illnesses. These infectious disease ICUs should have separate air conditioning vents and be located away from other high-risk areas of the hospitals. If this is not possible, ICUs should at least have isolation rooms with separate air conditioning vents. Additionally, like operation theatre staff, ICU staff should use surgical scrubs that must be donned in a designated donning area and taken off in a separate doffing area. The doffing areas should have showers and sanitisers so that the staff can shower before leaving the area.

Areas with a high prevalence of respiratory infections and HAIs can permanently adopt the strict mask adherence policies and hygiene protocols of the pandemic: staff should always wear surgical masks inside the ICU and wash their hands or sanitise them frequently, contact with patients in the ICU should be minimum and if needed, the HCWs must first wear gloves, which should be safely discarded immediately after handling the patient. Hospitals should categorise ICU surfaces into high-touch and low-touch surfaces, where high-touch surfaces should be sanitised more frequently than low-touch surfaces. Strict surface disinfection measures can also be continued throughout the hospitals to improve the hygiene standards of the healthcare facilities. Disposable equipment is preferred wherever possible, but when non-disposable equipment is unavoidable, it should be sanitised after each use. Additionally, the number of visitors to the ICU should be limited to only those necessary for patient care to protect both patients and others from unnecessary contact with any pathogen.

Finally, telemedicine should be utilised to its full potential globally. When fully exploited, we believe it can help better allocate resources and relieve excess medical workforce. The vast experience of older or retired physicians can be used without putting them at risk of exposure.

All these measures will make critical care more effective and safer and hopefully equip us to better control epidemics and pandemics in the future.

## Figures and Tables

**Table 1:** Summary of measures taken in handling patients with COVID-19 infection

Safety measures taken by hospital management for HCWs and patients	Air filtration measures for ICU	Generalised measures
Usage of face shields and surgical masks (6) PPE usage with separate donning (clean-filter) and doffing (contaminated-filter) areas with proper waste management and spaces for hand sanitisation (4, 13) Implementation of a ‘buddy system’ to keep a check on HCWs’ safety (13) Frequent temperature checking and submission, along with COVID-19 screening test for early infection detection and contact tracking (15) Restriction of interaction between infected and non-infected individuals after being tested positive for COVID-19 (4) Revision of visitors’ policies to ensure that only people essential for patient care visit and those with weak immunity, such as the elderly, are not allowed to visit (19)	Separation of COVID-19 ICU air conditioning vents from other ICUs and high-risk areas (4) 99% filtration effectiveness for > 5 mm particles and negative pressure spaces for < 5 mm particles (4) Turning off air conditioners with all windows kept open along with exhaust and blow-in fans in pre-existing ICUs (4) Specific AIIR for AGPs (9, 10) maintained at a negative pressure using HEPA filters attached to gas outlet linked to the atmosphere allowing air exchanging and filtration at least 6–12 times per hour (10–12) Designating separate rooms for critically ill COVID-19 patients (expensive measure), along with well-ventilated halls for ICU spaces and makeshift hospitals (cost-effective measure) (4)	Separation of COVID-19 ICU personnel and equipment from other parts of the hospital (12) Surface disinfection: High touch surfaces (frequently disinfected) and low touch surfaces (less frequently disinfected) (13) Frequent hand hygiene and shower practice before leaving hospital premises (13) Implementation of telemedicine to avoid COVID-19 exposure and reduce on-field burden of doctors (16, 17) Cleansing of non-disposable equipment using 70% alcohol or quaternary ammonium compounds (13) and sodium hypochlorite for cleaning surfaces during patient’s stay and after discharge (14) Usage of soap and sodium hypochlorite to remove air bubbles from exhausts (4)

Notes: HCW = healthcare workers; PPE = personal protective equipment; AIIR = airborne infectious isolation rooms; AGPs = aerosol generating procedures; HEPA = high-efficiency particulate air
